# Role of Macrophages and Related Cytokines in Kidney Disease

**DOI:** 10.3389/fmed.2021.688060

**Published:** 2021-07-08

**Authors:** Elena Cantero-Navarro, Sandra Rayego-Mateos, Macarena Orejudo, Lucía Tejedor-Santamaria, Antonio Tejera-Muñoz, Ana Belén Sanz, Laura Marquez-Exposito, Vanessa Marchant, Laura Santos-Sanchez, Jesús Egido, Alberto Ortiz, Teresa Bellon, Raúl R. Rodrigues-Diez, Marta Ruiz-Ortega

**Affiliations:** ^1^Cellular and Molecular Biology in Renal and Vascular Pathology Laboratory, Fundación Instituto de Investigación Sanitaria-Fundación Jiménez Díaz-Universidad Autónoma Madrid, Madrid, Spain; ^2^Red de Investigación Renal, Instituto de Salud Carlos III, Madrid, Spain; ^3^Renal, Vascular and Diabetes Research Laboratory, Fundación IIS -Fundación Jiménez Díaz, Universidad Autónoma, Madrid, Spain; ^4^Spanish Biomedical Research Centre in Diabetes and Associated Metabolic Disorders (CIBERDEM), Madrid, Spain; ^5^Laboratory of Nephrology and Hypertension, Fundación IIS-Fundación Jiménez Díaz-Universidad Autónoma Madrid, Madrid, Spain; ^6^La Paz Hospital Health Research Institute, Madrid, Spain

**Keywords:** macrophages, cytokines, kidney disease, CCL18, inflammation, CCL8

## Abstract

Inflammation is a key characteristic of kidney disease, but this immune response is two-faced. In the acute phase of kidney injury, there is an activation of the immune cells to fight against the insult, contributing to kidney repair and regeneration. However, in chronic kidney diseases (CKD), immune cells that infiltrate the kidney play a deleterious role, actively participating in disease progression, and contributing to nephron loss and fibrosis. Importantly, CKD is a chronic inflammatory disease. In early CKD stages, patients present sub-clinical inflammation, activation of immune circulating cells and therefore, anti-inflammatory strategies have been proposed as a common therapeutic target for renal diseases. Recent studies have highlighted the plasticity of immune cells and the complexity of their functions. Among immune cells, monocytes/macrophages play an important role in all steps of kidney injury. However, the phenotype characterization between human and mice immune cells showed different markers; therefore the extrapolation of experimental studies in mice could not reflect human renal diseases. Here we will review the current information about the characteristics of different macrophage phenotypes, mainly focused on macrophage-related cytokines, with special attention to the chemokine CCL18, and its murine functional homolog CCL8, and the macrophage marker CD163, and their role in kidney pathology.

## Introduction: Role of Immune Cells in the Onset and Progression of Kidney Disease

Renal inflammation arises as a protective response after kidney injury to fight against the initial insult and to establish tissue repair. However, if the reparative processes fail, this inflammatory response could be deleterious, participating in the kidney disease progression (1). This inflammatory response involves many different populations of immune infiltrating cells, including monocytes/macrophages, neutrophils, CD8+ and CD4+ lymphocytes, dendritic cells, mast cells and natural killer cells (2). Importantly, in chronic kidney disease (CKD), regardless of the underlying etiology, there is a persistent activation of the inflammatory response, characterized by immune cell recruitment throughout the kidney, leading to a local overproduction of growth factors and pro-fibrotic cytokines. This altered environment could activate different cellular and molecular processes, which cause progressive nephron loss and glomerular and interstitial fibrosis, leading to end-stage kidney disease and/or premature death (1–3). CKD is emerging as an important health problem due to the absence of early diagnostic biomarkers and effective treatments. Although many *in vitro* and experimental studies have extensively characterized the distinctive macrophage phenotypes in physiological and pathological conditions, the differences between human and murine macrophages complicate the extrapolation of preclinical results into human kidney disease, being a limitation on approaching macrophages as a therapeutic target to treat human CKD. Now, we review the macrophage characteristics and phenotypes, comparing human and mice data, and focused on novel macrophage-related cytokines as biomarkers or therapeutic targets for kidney disease. We will focus in two interesting candidates, the macrophage derived chemokine CCL18, as well as its murine functional homolog *Ccl8*, and the macrophage marker CD163.

## Macrophage Characteristics and Phenotypes

Macrophages are innate immune cells and the main component of the mononuclear phagocyte system. Therefore, they are crucial in host defense against pathogens. Furthermore, macrophages are present in almost every organ in adult mammals, where they participate in multiple cellular processes and play a major role in the maintenance of tissue homeostasis (4). Macrophages are considered as tissue sentinels that maintain tissue integrity by eliminating senescent and dead cells, and debris (5). Moreover, they also participate in extracellular matrix (ECM) remodeling, mainly through matrix metalloproteinases (MMPs) release (6), and in the restoration of lost cells and intercellular matrices through the production of various regenerative growth factors (4).

Tissue resident macrophages (TRMs) can be classified into two different subtypes depending on their origin; one derived from circulating monocytes (7–9), and the other from embryonic precursors that are able to locally proliferate and self-renew (10, 11). Monocytes are limited to the blood compartment, the spleen and the bone marrow (12), but in response to inflammation, and guided by the cytokine milieu and/or interactions with other cells and microbial products, monocytes are quickly recruited into injured tissues and then differentiate into several specific macrophage phenotypes depending on micro-environmental signals (8, 13).

### Macrophage Polarization: M1 and M2 Phenotypes

**Figure 1:**
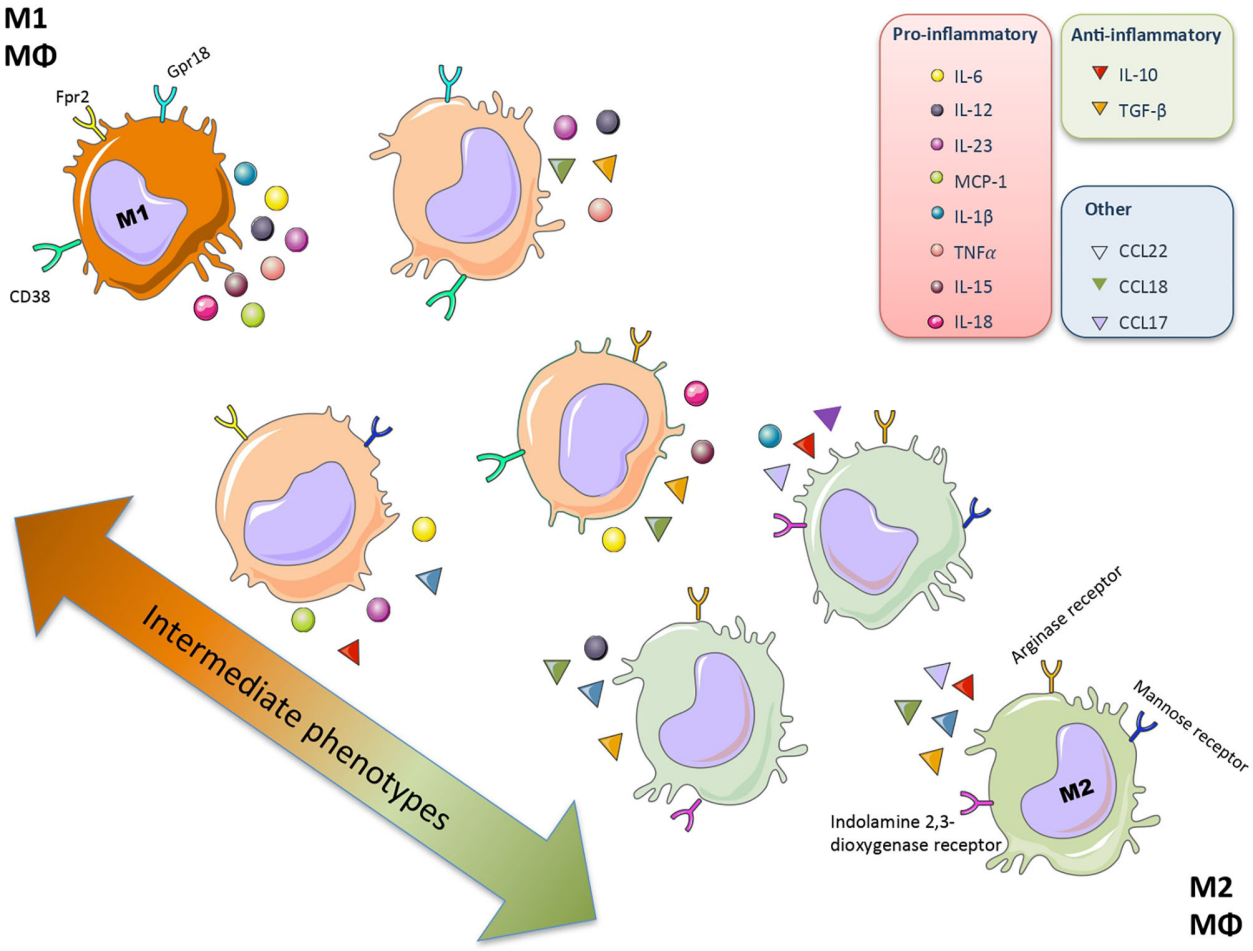
Macrophage polarization. Macrophages (Mθ) have a great plasticity and have different functional activation states and phenotypes that allow different specialized functions. The macrophage population represents a continuous phenotype ranging between M1/M2 extremes, characterized by a specific secretome (mainly cytokine and chemokines) and surface markers.

**Figure 2:**
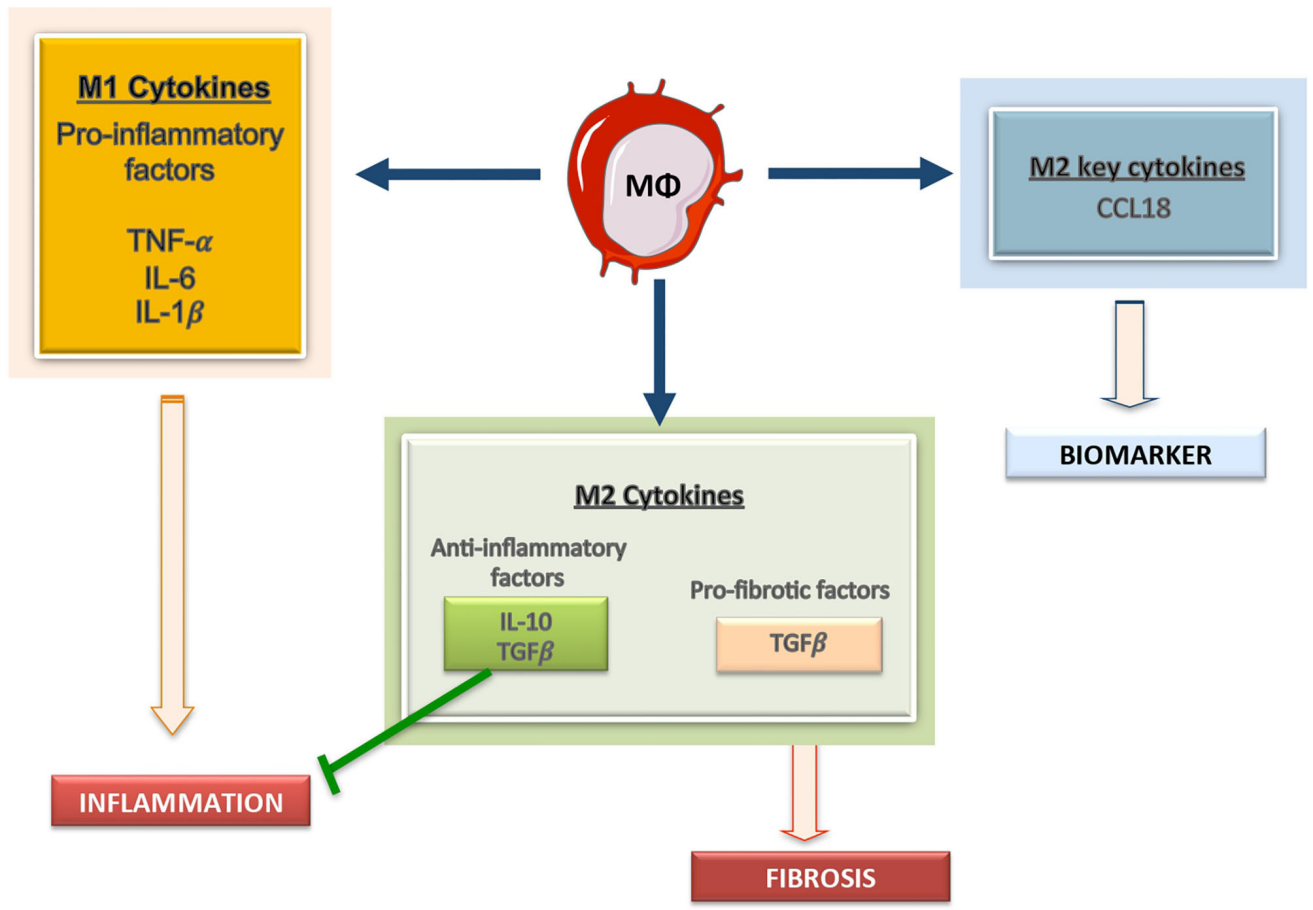
Macrophages derived cytokines can contribute to kidney damage. Macrophages can secrete a large array of molecules, including producing pro-inflammatory mediators, including IL1, Il6, and TNF, anti-inflammatory cytokines, such as IL-10, and profibrotic factors, like TGF-β. Among the M2 macrophage related factors CCL18 is a recent described cytokine and a potential biomarker of disease progression.

Plasticity is the hallmark feature of macrophages. The term “macrophage polarization” is used to refer to an estimate of macrophage activation status and phenotype (11). The M1/M2 polarization axis was originally defined in the 1990s (14) to describe the dichotomy in macrophage function regardless of cytokines: classically activated macrophages/M1 microbicidal macrophages and alternatively activated macrophages/M2 macrophages (Figure 1). M1 macrophages participate in the infection clearance and act as an initial defense barrier. The M1 phenotype is generated in response to pro-inflammatory stimuli, such as pathogen/danger-associated molecular patterns (PAMPs or DAMPs) in the presence of interferon gamma (IFN-γ) (14, 15). M1 cells are characterized by their ability to secrete significant amounts of pro-inflammatory cytokines including tumor necrosis factor alpha (TNF-α), interleukin-1β (IL-1β), interleukin-6 (IL-6), interleukin-12 (IL-12), interleukin-15 (IL-15), interleukin-18 (IL-18), and interleukin-23 (IL-23) (16–18). M2 macrophages can be a “two-edged sword.” On one side, the anti-inflammatory phenotype is essential for adequate tissue repair; on the other side, it is a potential mediator of fibrosis and scarring (15). These M2 macrophages can release a different cytokine profile, such as transforming growth factor-β1 (TGF-β1), interleukin-10 (IL-10), C-C Motif Chemokine Ligand 17 (CCL17), C-C Motif Chemokine Ligand 18 (CCL18) and C-C Motif Chemokine Ligand 22 (CCL22) (Figure 2) (19–24). The macrophage population is currently thought to represent a continuous phenotype ranging between M1/M2 extremes and including other ill-defined populations, as commented above (Figure 1). Moreover, the M1/M2 classification is not representative of *in vivo* events, given that M1 and M2 stimuli do not exist alone in tissues. While a much broader spectrum of innate immune responses has been characterized, the M1/M2 axis remains the main macrophage polarization axis *in vivo* (25).

Most of the earliest studies on macrophage polarization were based on *in vitro* stimulation with different combination of cytokines. One of the most important regulators of macrophage differentiation is the colony-stimulating factor 1 receptor (CSF1R). This is a transmembrane tyrosine kinase receptor expressed on almost all mononuclear phagocytic cells (26). *Csf1r* gene deletion depleted macrophages in several tissues. Although this study demonstrates its key role in macrophages development, it also showed that some macrophages were still present, indicating the involvement of other growth factors in macrophage differentiation (27), including Granulocyte-macrophage colony-stimulating factor (GM-CSF), interleukin-3 (IL-3), vascular endothelial growth factor (VEGF), and fms-like tyrosine kinase 3 ligand (Flt3L) (28–30). Macrophages respond in different ways to macrophage colony-stimulating factor (M-CSF) and GM-CSF with substantial polarizing effects. These factors have been used to study *in vitro* differentiation/polarization of macrophages (31–33). In this sense, GM-CSF activates the inflammatory program and extensive DNA methylation changes, while M-CSF-polarized cells (less differentiated state) show characteristics of anti-inflammatory repairing macrophages (33). Further research led to a sub-classification of M2 macrophages into M2a, M2b, and M2c described as wound-healing, immune complexes-related or regulatory macrophages, respectively (11, 16, 34). M2a activation induced by IL-4 or IL-13 was associated to changes in surface markers, including decreased CD14 expression, and upregulation of mannose receptor (CD206), CD209, and CD23. In M2a macrophages, pro-inflammatory cytokine expression is lower than in M1, but they release several factors, including CCL18 and vascular Endothelial Growth Factor (VEGF) (35). M2a can also secrete ECM components, like soluble fibronectin, and therefore their primary function is thought to be related to wound healing and tissue remodeling and repair (36). M2b cells are elicited *in vitro* by stimulation with LPS or IL-1β plus high concentrations of immune complexes. M2b macrophages are considered as functional converter of M1 cells with low IL-12 and high IL-10 production. Interestingly, they selectively produce the C-C Motif Chemokine Ligand (CCL1) (37). The M2c category includes cells stimulated with IL-10, TGF-β1 and glucocorticoids, being a heterogeneous group, but characterized by high expression of the surface marker CD163 (38). RNAseq studies have identified M2c-specific genes associated with angiogenesis, matrix remodeling, and phagocytosis, including CD163 (39). Accordingly, the analysis of the M2c macrophage-conditioned media revealed elevated production of MMPs (39). Due to the controversies in the field, a specific nomenclature for cytokine-induced macrophage polarization *in vitro* M (cytokine) have been proposed (40).

Originally, the distinction between M1 and M2 phenotypes was also based on differences in arginine metabolism (14), but recent research has unraveled the metabolic differences associated with macrophage phenotype switching (41). As an overview, and based on the premise that M1 macrophages kill pathogens and M2 resolve inflammation and tissue repair, M1 are more likely to be involved in catabolic pathways, and M2 in anabolic ones (42). Therefore, M1 tend to rely on working aerobic glycolysis likewise fatty acid biosynthesis, because of the speed of producing ATP, but it also have impaired oxidative phosphorylation (43). Moreover, in M1 macrophages, nitric oxide synthase is highly expressed, playing an essential role in the M1 function of killing pathogens, being nitric oxide the source of reactive oxygen species (ROS) with antimicrobial properties (36, 44). On the other hand, M2 are more suitable to trigger fatty acid oxidation as energy-production pathway (42). These macrophages secrete insulin-like growth factor 1 (IGF-1) (45), TGF-β1, and VEGF (46), as well as use arginine to produce precursors of collagen (47), thereby dampening inflammation.

Though we are aware that it may represent an oversimplification, since most of the previous literature has used the M1/M2 classification nomenclature, this terminology has been maintained in some parts of this review.

### Comparison of Human and Mammalian Macrophages: Limitations, Similarities, and Future Perspectives

A limitation in the field of macrophage research is the extrapolation of preclinical studies to humans. Macrophages play a key role in the inflammatory response, but the pathogen type that infects humans can be different from other species, including rodents, as well as the molecules released by macrophages to control infections.

The studies investigating M1/M2 macrophage polarization, some of them included in the previous section, were done using mouse or human macrophages of different cell sources; including isolated circulating monocytes, bone marrow-derived cells or peritoneal macrophages, as well as established cell lines (such as THPs or RAW 264.7). A macrophage comparative study evaluating different cell sources depicted similarities between mouse and human cytokine profiles stimulated with a specific combination of cytokines, such as CXCL-10 and CXCL-11 for M (LPS,IFN) and CCL17 and CCL22 for M (IL4, IL13)-induced macrophage polarization (48). Although murine M1/M2-polarized macrophage subsets can be distinguished on the basis of combinatorial gene expression profiles, the identification of equivalent subsets in humans is still unresolved. Another key point is the specific M1/M2 macrophage subsets markers. In humans, similarly to mice, three monocyte subsets have been described according to differential expression of CD14 and CD16 on HLA-DR^+^ cells (49). In human macrophages, CD68 is a general marker, whereas HLA-DR and CD163 are M1 and M2 markers, respectively (49, 50). Murine and human macrophages express the antigens CD68, CD11b, CSF1R, and CD163 (5). F4/80 is expressed on most tissue macrophages in the mouse and it has been extensively studied by immunohistochemistry, but has limited usefulness in humans as F4/80 is predominantly expressed on eosinophils (51). Recent research is focused on the search of additional markers. The early growth response protein 2 (Egr2) and c-Myc have been described in murine models to identify *in vivo* polarized M2 cells. One the other hand, some *in vivo* pro-inflammatory factors have been shown to be M1 macrophage markers, such as G-protein-coupled receptor 18 (Gpr18), formyl peptide receptor 2 (Fpr2), CD40, and CD38 (52, 53). Among them, CD38 could be also a marker of M1 pro-inflammatory macrophages in humans (54). Comparative biology, together with omics technologies, such as transcriptomics, metabolomics, proteomics, and epigenomics could potentially be employed to assess similarities between mouse and human macrophages (4). Recently, the single-cell RNA sequencing across multiple mammalian species has led to the identification of CD74 and CD81 as surface markers for kidney resident macrophages (55). Future studies in human biopsies of different pathologies using cutting edge technologies will reveal human macrophages characteristics in each state of the disease.

## Macrophages in Renal Diseases: Phenotypes and Functions

Macrophages participate in immune surveillance and in the regulation of kidney homeostasis. The macrophage response to kidney injury varies enormously depending on the nature and duration of the insult (23). Macrophages participate in the inflammatory response, both in acute kidney injury (AKI) and in CKD. They can promote kidney repair or contribute to the AKI-to-CKD transition and fibrosis, highlighting their remarkable plasticity (2, 56).

### Macrophages in AKI: From Preclinical Data to Human Studies

Macrophages can actively participate in all AKI-related processes, including cell death, resolution/regeneration phase or progression to kidney fibrosis (57). Importantly, macrophage phenotypes can change along AKI, depending on disease stage and evolution. Circulating classical monocytes (CD11b+ Ly6C^high^ in the mouse or CD14^high^CD16^low^ in humans) are recruited into the kidney, where they differentiate to pro-inflammatory M1 macrophages during the early phase of renal injury, in response to infection or cell damage (23). Many preclinical data suggest that M1 macrophages play a pathogenic role in the early phases of AKI. Studies done in the model of folic acid in mice showed an upregulation of cytokines and chemokines, such MCP-1/CCL2 (the main macrophage chemotactic factor) associated to the presence of M1 macrophages in the injured kidney at 48 h (58). Moreover, in ischemia-reperfusion injury (IRI) in mice M1 macrophages, identified by their high expression of iNOS, IL-12, IL-23, and Ly6C, were detected also at 48 h (59). Accordingly, depletion of kidney macrophages by liposomal clodronate (LC) at the early stages of IRI reduces AKI and improves renal repair. Furthermore, adoptive transfer of IFN-γ-stimulated macrophages in LC-treated IRI mice worsens AKI (49). Following AKI, once pathogens or injured cells are cleared up, a rapid polarization of macrophage phenotypes is necessary for tissue regeneration. During the regeneration process there is a restoration of the polarized tubular epithelium and basement membrane integrity, together with neovascularization, ensuring the recovery of tissue oxygenation by the injured microvasculature, leading to the reestablishment of the tubular cell functionality (60, 61). Therefore, a decrease in infiltrating M1 macrophages is essential to minimize injury of surrounding cells, since macrophages release cytotoxic compounds that do not distinguish self from exogenous pathogens (62, 63). Preclinical studies have described high levels of IL-4, IL-10, and IL-13, leading to a macrophage switch to M2 phenotype characterized by high expression of arginase-1 (Arg1), mannose receptor (MR, also termed CD206), chitinase-like protein (e.g., Ym1), resistin-like protein (Fizz1) and CD36 (fatty acid translocase), associated with down-regulated expression of proinflammatory markers (i.e., IL-12 and iNOS) (49). On the other hand, the prolonged presence of infiltrating macrophages in the kidney might be associated with persistent release of wound-healing growth factors, such as TGF-β1. This may turn the initial wound healing process to pathological one, resulting in further tissue damage, and contributing to the AKI-to-CKD progression, and irreversible fibrosis (64). Nevertheless, data on human macrophage phenotypes and related cytokines in healthy and AKI kidneys have been scarcely studied, limiting the translation of preclinical studies to humans. Moreover, the mechanisms enabling macrophage switch from the M1 to M2 subset remain unclear (49). Studies of human AKI biopsies have identified macrophages as the main cell type infiltrating the kidney that persist during tissue repair, being CD163 expressing -macrophages the predominant phenotype in the late phase of AKI (50, 61). Future studies in human AKI at different disease stages are needed.

### Macrophages in CKD

Kidney infiltration by macrophages is common in human CKD. The magnitude of macrophage infiltration correlates with the severity of kidney injury suggesting an effector function of macrophages in CKD (19). However, the role of M1/M2 macrophages in human CKD progression is still poorly understood. In experimental progressive CKD, M1 macrophages are present in the early phases of inflammation (23) and, as this process progresses, M2 macrophages predominate to encourage repair and/or fibrosis (65), as described in unilateral ureteral obstruction (UUO) model (57, 66, 67). Some studies have suggested that the M1/M2 macrophage balance could influence CKD development (68, 69). Both M1 and M2 responses coexist during CKD (68). Indeed, M2 macrophages could originate from M1 macrophages or from proliferation/differentiation of monocytes (23, 65, 68). Some evidences suggest that macrophages can directly promote kidney fibrosis. The CD206^+^ subset of M2 macrophages is strongly associated with kidney fibrosis in both human and experimental diseases (23). Indeed, bone marrow-derived M2-type pro-fibrotic macrophages are highly proliferative, which may contribute to promote kidney fibrosis in experimental models such as UUO and the Adriamycin nephropathy (70–72).

### Macrophages in Hypertension and Related Kidney Damage

In experimental hypertension, M2 macrophages expressing Mouse Chitinase 3-like 3(Chi3l3)/YM1were associated with renal fibrosis (73). Macrophages may promote hypertension through the generation of M1 cytokines, such as TNF-α, IL-6, and IL-1β, and reactive oxygen species (ROS) resulting in enhanced renal sodium retention and organ damage (74, 75). In this sense, recent studies suggest that the pro-inflammatory cytokine IL-17A, produced by CD4^+^/T and γδ-lymphocytes, but by not macrophages, plays a key role in the onset of hypertension and in hypertensive end-organ damage, such as the heart, vessels and kidneys (76, 77). However, the Th17 phenotype is sustained by interleukin-23 (IL-23), produced mainly by M1 macrophages (16), showing an interplay between macrophages and immune cells in hypertension.

## Novel Macrophage-Derived Factors as Potential Biomarkers in CKD

Macrophages can release a wide array of cytokines, which varies depending on pathological conditions. One well-known product of macrophages is MCP-1/CCL2, a chemokine driving their recruitment into injured tissues (78). Some clinical data suggest that MCP-1/CCL2 could be a biomarker of kidney fibrosis and function decline (79, 80). As commented above, M1 macrophages can produce TNF-α, IL-1β, IL-6, IL-12, IL-15, IL-18, and IL-23 whereas M2 macrophages release TGF-β1, IL-10, CCL17, CCL18, and CCL22 (Figure 2). Therefore, macrophage-derived biomarkers in blood and other biological fluids can reflect the activation of macrophage populations in tissues. In this sense, IL-6 is now considered an important cardiovascular risk biomarker (81). Some of these macrophage-derived cytokines can be relevant as biomarkers of kidney disease progression. Additionally, in the AKI-to-CKD transition, the evaluation of M1/M2 macrophage markers or secretome-derived factors can be used to monitor disease progression and/or remission (23). We will now focus in two interesting candidates, the macrophage derived cytokine CCL18, and the macrophage marker CD163.

### CCL18 and Macrophage Functions

Chemokine (C-C motif) ligand 18 (CCL18) regulates several inflammatory and immunological processes, participating in cell recruitment (82, 83) and in phenotype transformations in cancer cells (84). This chemokine is constitutively expressed in the lung and in antigen presenting cells, such as dendritic cells and keratinocytes (83). CCL18 is one of the most highly expressed chemokines in human chronic inflammatory diseases, including allergies, fibrotic disorders and certain cancers (83). As commented before, M2 macrophages secrete high amounts of CCL18 (35). Moreover, stimulation of monocyte/macrophages with CCL18 induces an M2 spectrum macrophage phenotype (20).

#### CCL18 as Biomarker of Disease Progression

In some diseases, CCL18 levels are used as a biomarker of disease progression, for instance in Gaucher disease (85) idiopathic pulmonary fibrosis (86), and chronic periaortitis (87), as well as in several proliferative disorders, including breast (88) and lung (89) cancer, glioblastoma (90), bladder cancer (91), osteosarcoma (92), and prostate cancer (93, 94). However, there is scarce information in kidney diseases. Serum CCL18 levels were proposed as a biomarker of disease activity in ANCA-associated crescentic glomerulonephritis (95). In patients with CKD undergoing peritoneal dialysis treatment, CCL18 levels in peritoneal effluent correlated with progressive ultrafiltration failure and peritoneal fibrosis, suggesting that CCL18 could also be a biomarker of peritoneal damage (96).

#### Mouse CCL8 Is the Functional Analog of Human CCL18 and Shares CCR8 as Functional Receptor

The human CC chemokine receptor 8 (CCR8) is a seven-transmembrane-spanning G protein–coupled receptor, whose canonical ligand is Chemokine (C-C motif) ligand 1 (CCL1/I-309) (97). CCL18 was recently discovered as another CCR8 agonist with less affinity than CCL1 (98). CCR8 is expressed mostly in monocytes and thymus and acts as chemoattractant receptor for Th2 cells (99). Moreover, this receptor was found in lymphocytes in human healthy skin for preserving tissue homeostasis (100). However, CCR8 is not expressed in human kidneys in normal or pathological conditions, such as renal transplant rejection (101). In contrast, CCR8 expression was detected mainly in tumor renal cells of human renal cell carcinoma (102). Accordingly, upregulated expression of CCR8 has been described in circulating cells of patients with bladder and renal carcinoma (103).

Human CCL18 and mouse chemokine (C-C motif) ligand 8 (CCL8), named here as mCCL8, have been proposed to be functional analogs (98). The CCL18 gene has only orthologs in primates (104), whereas mouse *Ccl8* gene (also known as *Mcp-2*) lacks a human ortholog as showed by phylogenetic analysis and synteny mapping, but binds to CCR8 (105), and shares functions with CCL18 (98). Importantly, human CCL8/MCP2 is a different cytokine, exerting chemotactic actions for macrophages, and signaling through CCR1 and CCR5 (106). Therefore, to avoid confusion along the manuscript we have named mouse CCL8 here as m*Ccl8*.

#### Human CCL18 and Mouse CCL8 in Human and Murine Kidney Disease, Respectively

**Figure 3:**
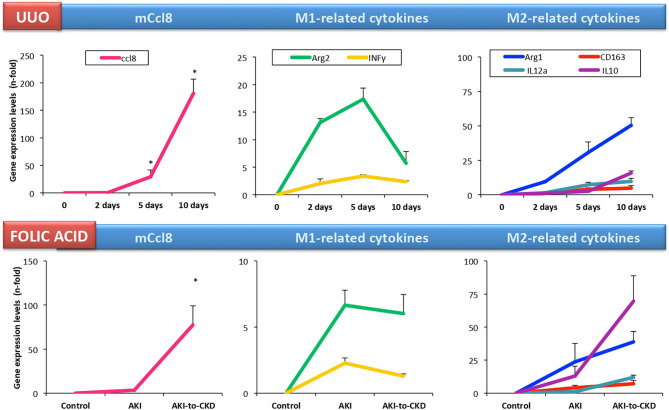
Kidney *Ccl8* gene expression is associated to M2 cytokines in experimental kidney injury. Kidney *Ccl8* expression and M1 and M2-related factors were evaluated in CKD models: unilateral ureteral obstruction (UUO) and the acute and chronic phase of folic acid (FA) nephropathy mice [data from Cantero-Navarro et al. (110)].

A microarray analysis of renal biopsy samples of patients with newly diagnosed ANCA-associated crescentic glomerulonephritis identified CCL18 as the most upregulated gene (95). Immunohistochemical analysis identified myeloid dendritic cells and CD68^+^ macrophages as CCL18-producing cells, and determined that the density of CCL18^+^ cells correlated with interstitial inflammation, crescent formation and impairment of renal function at the time of biopsy. Serum CCL18 levels also correlated with kidney disease activity, being lower in patients with immunosuppressive rescue therapy and higher in relapsing kidney disease (95). In accordance to these data, we found that CD163^+^/CCL18 expressing macrophages colocalized with Gremlin protein expression in another cohort of patients with ANCA-associated crescentic glomerulonephritis (107). In this study, we proposed urinary Gremlin levels as a potential biomarker of disease progression, showing a parallelism to CCL18. Despite these comprehensive studies, there are no published data on CCL18 in other human kidney diseases. In cultured human tubular cells, CCL18 increased the production of fibronectin in diabetic conditions (108). On the other hand, there is scarce information about the role of mCCL8 in preclinical kidney disease. In murine renal artery stenosis, kidney m*Ccl8* gene expression was higher than in control mice (109). However, in cultured murine tubular cells, we have found that these cells lack *Ccr8* expression and were not responsive to mouse recombinant CCL8 protein (110). In experimental folic acid-induced AKI, kidney m*Ccl8* expression was unchanged in the acute phase (24–72 h). However, kidney m*Ccl8* expression was upregulated at 7 days, a time point associated to the AKI-to-CKD transition. In UUO, kidney m*Ccl8* expression was already increased at 5 days (111), and increased progressively over time (Figure 3). The evaluation of M1/M2 cytokine profiles showed that kidney m*Ccl8* expression correlates to M1-related cytokine downregulation and M2 cytokine overexpression. Therefore, these data suggest a potential role of m*Ccl8* in mouse macrophage polarization toward the M2 phenotype, helping to maintain chronic inflammation and favoring kidney fibrosis.

We previously showed that increased M2 macrophage numbers associated to peritoneal CCL18 effluent concentrations in patients with CKD undergoing peritoneal dialysis (96). Moreover, in a murine model of exposure to peritoneal dialysis fluids (112) we have found that m*Ccl8* expression was increased associated to macrophage infiltration (113), supporting the concept of mouse *Ccl8* as a functional analog of human CCL18.

### CD163 in Kidney Injury

CD163 is a glycosylated membrane protein, member of the scavenger receptor cysteine-rich family, also known as the hemoglobin scavenger receptor (13, 114, 115). CD163 is expressed nearly exclusively by cells of monocytic origin (e.g., monocytes, macrophages, some dendritic cells, and some tumor cells) presenting high expression levels in macrophages (13, 114, 115). By ectodomain shedding, the extracellular portion of CD163 can be released from the cell surface into circulation, in a process regulated by ADAM17/TACE (tumor necrosis factor α-converting enzyme) (116). Soluble CD163 (sCD163) is stable and measured easily in serum, and has been investigated as potential biomarker of macrophage activation in various disease contexts (117–119).

CD163 functions have been extensively reviewed (120, 121). Early studies described that CD163 expression is a hallmark of the wound healing macrophage, related to the resolution phase of inflammation after cardiopulmonary bypass surgery (122) and in human inflammatory skin disease induced by Cantharidin (123). Some studies in animal models of renal damage pointed out the key role of CD163 in the beginning and progression of renal disease (124–126). In AKI induced by experimental rhabdomyolysis increased levels of M1 macrophages were observed in the early pathological stages. These macrophages suffer a partial differentiation to M2 phenotype characterized by CD163 overexpression via HO-1 activation and IL-10 release. Moreover, peritoneal macrophages stimulated by myoglobin can induce fibrosis through the regulation of profibrotic mediators, such as TGF-β and CTGF/CCN2 (124). In cisplatin-induced nephrotoxic damage in rats increased levels of CD163 M2 macrophages associated to fibrosis were found (126). Other study in Lupus nephritis (LN) in mice described an increased CD163^+^/CD68^+^cell ratio. In addition, *Cd163* gene expression was increased in LN mice while *Ho-1*, levels were reduced, the last one associated to elevated *Bach1* and *Il-6* expression. The gene blockade of *Bach1* in mice (Bach1-deficient MRL/lpr mice) improved the loss of renal function in experimental LN assessed by BUN levels (125).

*In vitro* studies in human macrophages found that CD163 overexpression induces a change on the cytokine profile secretion from pro-inflammatory M1-related cytokines to M2-cytokines (127). Importantly, CD163^+^/CD68^+^ macrophages may be involved in the pathogenesis of proliferative glomerular crescents, such as in ANCA-associated glomerulonephritis or active LN. CD163^+^/CD68^+^ macrophages were found in glomerular crescents and were correlated to proteinuria and to estimated glomerular filtration rate (eGFR) (positively and negatively, respectively) (128). Moreover, two different M2 macrophages populations, CD163^+^ and CD206^+^, were mainly expressed in fibrous crescents and were more common in Lupus nephritis (LN) and ANCA-associated vasculitis than in IgA nephropathy and Henoch Schönlein purpura glomerulonephritis (129, 130). Patients with early stage of idiopathic membranous nephropathy had higher levels of circulating CD14^+^/CD163^+^, CD14^+^/CD163^+^/CD206^+^, and CD14^+^/CD163^+^/CD206^+^/CD115^+^ macrophages in comparison with healthy controls (131). M2-macrophages were considered the dominant subpopulation in human LN and M2a subpopulations were associated with disease progression (132). In ANCA glomerulonephritis, urinary CD11b^+^ and CD163^+^ correlated with leukocyte recruitment in the kidney (133). Indeed, urinary sCD163 is a marker of human glomerulonephritis (118, 134) and active renal vasculitis (135, 136). On the other hand in LN (119), sCD163 in the urine is considered as a marker of disease activity and treatment response (137, 138). Interestingly, CD163^+^ cells in crescents of ANCA glomerulonephritis patients colocalized with CCL18 (95) and Gremlin (107). Both CCL18 and Gremlin levels have been independently proposed as potential biomarkers of disease progression (95, 107). These two independent studies suggest that the M2 macrophage secretome could be a source of biomarkers of kidney disease progression, mainly in crescentic glomerulonephritis. Accordingly, in peritoneal dialysis patients, CD163^+^ macrophages were present in peritoneal effluents and increased during peritonitis. Moreover, peritoneal CCL18 effluent concentrations correlated with decreased peritoneal function, showing the contribution of macrophage-derived cytokines to peritoneal damage and fibrosis (96). Despite there are few data about sCD163 in human diabetic nephropathy some studies described that sCD163 levels were also strongly associated with later development of type 2 diabetes in both lean and obese subjects, likely reflecting macrophage recruitment in the adipose tissue (117, 139–143) and in the liver (144–146), an effect associated to ADAM17/TACE-mediated shedding of TNF-α and sCD163 (116). Diabetic patients had higher numbers of circulating CD163^+^ monocytes (147). In addition, sCD163 was also identified as a good risk biomarker of diabetic nephropathy and/or diabetic retinopathy (148). Anti-inflammatory CD163^+^ macrophages were elevated in glomeruli from diabetic patients and were associated with pathological features such as tubular atrophy, interstitial fibrosis and glomerulosclerosis (149). In murine RAW264.7 macrophages preincubated with high glucose, calcitriol (1,25(OH)_2_D_3_) treatment blockaded M1 macrophage activation and M2 phenotype differentiation. The same result was observed in streptozotocin (STZ)-diabetic rats treated with calcitriol (150). Moreover, the uremic toxins that are accumulated in the last stage of renal disease have a role in M2 induction. A study in THP-1 cells showed that indoxil sulfate (IS) induced CD163 expression and transition to macrophages through AhR/Nrf2 activation (151). Finally, other study linked the increase of fat mass with elevated levels of sCD163, suggesting that adipose tissue macrophages play a key role in CKD proinflammatory state (140).

sCD163 was also been found in heme-related human kidney injury, such IgA nephropathy (152), intravascular hemolysis (e.g., paroxysmal nocturnal hemoglobinuria) (153), favism (154), and rhabdomyolysis AKI (124). In macrophages of patients with IgA nephropathy and macroscopic hematuria-related AKI, CD163 was associated with incomplete recovery of kidney function (152, 155) describing as the predominant subpopulations in kidney tissues, the M2a (CD206+/CD68+) and M2b (CD86+/CD68+) macrophages (130).

## Targeting Macrophage Related Pro-Inflammatory Cytokines as Therapeutic Approach for Kidney Disease

There have been multiple attempts to inhibit or modulate the inflammatory response to prevent or retard CKD progression. The most widely used approaches to modulate macrophage levels and/or phenotypes have been directed to M1-related cytokines, such as IL1, IL-6, and TNF-α. We now discuss preclinical studies targeting those inflammatory mediators and the key monocyte/macrophage chemotactic factor MCP-1/CCL2, and the potential translation to humans.

### Interleukin-1 Blockade in Experimental and Human Kidney Disease

The earliest studies targeting macrophage related cytokines tested IL-1 strategies. Treatment with an IL-1 receptor (IL-1R) antagonist (IL-1Ra) ameliorated experimental anti-glomerular basement membrane antibody-associated glomerulonephritis (anti-GBM) in rats (156) as well as spontaneous IgA nephropathy in ddY mice (157). Gene deletion of IL-1 Type 1 Receptor (IL1R1) or IL-1β demonstrated that IL-1β but not IL-1α contributed to crescent formation and inflammatory cell recruitment in murine anti-GBM crescentic glomerulonephritis (158). Similarly, genetic IL1R1 deletion modestly improved survival and attenuated cyst volume in experimental murine ADPKD (159). The IL-1R1 antagonist anakinra prevented nephropathy in diabetic mice (160). Accordingly, anti-IL-1β antibody attenuated the progressive loss of kidney function and preserved podocytes in murine loss of kidney mass in diabetic *db/db* mice (161). Human studies also showed beneficial effects, including improved vascular endothelial function in patients with non-dialysis-dependent CKD after 12 weeks of treatment with the IL-1 inhibitor rilonacept, whereas no changes in kidney function were observed nor expected in such a short follow-up (162). A clinical trial of the canakinumab (neutralizing antibody against IL-1β) observed a reduction in the cardiovascular event rates in atherosclerosis patients with CKD without modifying kidney function (163). However, a clinical trial of gevokizumab (antibody against IL-1β) in diabetic kidney disease was terminated prematurely, based on company priorities (164).

### TNF-α Blockade

There are multiple preclinical examples of kidney protection afforded by anti-TNF strategies. Administration of a pegylated form of the soluble TNF type 1 receptor (PEG-sTNFR1) reduced renal fibrosis in experimental rat CKD induced by renal mass reduction (165) and kidney inflammation and tubular cell apoptosis in rat UUO (166). Similarly, TNF-α gene deletion or silencing attenuated kidney injury induced by high fat diet in mice by reducing fibrosis and glomerulosclerosis (167). A neutralizing anti-TNF-α antibody reduced glomerular inflammation, crescent formation, and tubulointerstitial scarring, and preserved kidney function in rat anti-GBM crescentic glomerulonephritis (168). An anti-TNF-α antibody decreased albuminuria, plasma creatinine, histopathologic changes, and kidney macrophage recruitment in an experimental type I diabetes in B6-Ins2Akita/MatbJ mice (169). Moreover, macrophage-specific TNF-α-deficient mice (CD11b^Cre^/TNF-α^Flox/Flox^; C57BL/6) presented the same beneficial effects after streptozotocin-induced diabetes (169). In high fat diet-induced kidney injury, TNF-α deletion reduced kidney fibrosis, glomerulosclerosis, oxidative stress, inflammation and apoptosis (167). Despite promising preclinical results, targeting TNF-α in human kidney disease for kidney protection is controversial. A clinical trial of infliximab in LN failed in the recruitment phase (NCT00368264). However, anti-TNF-α monoclonal antibodies are used routinely to treat rheumatoid arthritis (RA), ankylosing spondylitis (AS) or psoriasis. Thus, there is mixed information on such patients and kidney disease. Some reports observed that in RA patients with CKD, anti-TNF-α drugs (adalimumab, etanercept, or infliximab) had no deletereous effect on kidney function (170) or presented slower loss of renal function (171). However, cases of AKI, focal segmental glomerulosclerosis (FSGS) or IgA nephropathy have been reported in AS, RA, or inflammatory Bowel Disease patients treated with anti-TNF-α drugs (172–176).

### IL-6 Blockade in Experimental and Human Kidney Disease

Due to the key role of IL-6 in kidney diseases (177), the impact of targeting IL-6 has been studied. However, preclinical results differed for some kidney diseases. Neutralizing IL-6 (178), IL-6 receptor (IL-6R) (179) or genetic IL-6 deletion (180) decreased disease severity in different experimental models of LN in MRL-Fas^lpr^ and NZB/WF1 mice. However, anti-IL-R6 or anti-IL-6 strategies increased the severity of murine anti-GBM nephritis, while selective inhibition of IL-6 trans-signaling by sgp130Fc did not (181). Similarly, genetic IL-6 deletion did not decrease fibrosis in murine UUO (182), whereas treatment with Fc-gp130 reduced inflammation, immune cell infiltration and fibrosis in both murine UUO and IRI (65). These findings fit well with the concept that IL-6 classic signaling is anti-inflammatory and protective, whereas IL-6 trans-signaling is pro-inflammatory.

In addition to inflammation, IL-6 controls glucose metabolism and the hypothalamic-pituitary-adrenal axis among other processes which could result in deleterious effects when IL-6 is modulated (183). Therapies modulating IL-6 (olokizumab or clazakizumab) or the α subunit of its receptor (tocilizumab or sarilumab) have been used clinically to treat inflammatory diseases, such as RA, systemic lupus erythematous (SLE), diabetes and, more recently, coronavirus disease 2019 (COVID-19) (184, 185) that may develop kidney disease, but no clinical trial studied kidney function or renal injury as primary outcome. An ongoing trial is exploring the efficacy of clazakizumab to preserve kidney function in highly sensitized patients awaiting kidney transplantation (NCT03380962) or in kidney transplant receptors with late antibody-mediated rejection (NCT03444103).

### MCP-1/CCL2 Blockade

MCP-1/CCL2 is the main factor driving monocyte recruitment and differentiation during inflammatory response. Therefore, MCP-1/CCL2 has been targeted in preclinical kidney disease (186). Both genetic deficiency of the MCP-1 receptor (CCR2) or MCP-1/CCL2 antagonism improved LN and vasculitis in MRL/lpr mice (187, 188). Likewise, MCP-1/CCL2 neutralizing antibody reduced glomerular macrophage infiltration and decreased crescent formation in experimental rat and murine crescentic glomerulonephritis (189, 190). Targeting MCP-1/CCL2/CCR2 was also protective in experimental diabetic nephropathy. A CCR2 antagonist (propagermanium) protected the kidneys in type 1 diabetic mice overexpressing type 2 nitric-oxide synthase (191), MCP-1/CCL2 gene deletion decreased glomerular and interstitial macrophage accumulation and fibrosis in murine STZ-induced diabetic nephropathy (192), and the MCP-1/CCL2 antagonistic Spiegelmer mNOX-E36 (emapticap pegol) reduced the number of glomerular macrophages and improved glomerular filtration rate (GFR) in uninephrectomized *db/db* mice (193) and in STZ- diabetic ApoE knockout mice (194). However, in experimental murine Alport nephropathy (Col4a3-deficient mice), mNOX-E36 administration reduced glomerular and interstitial macrophage recruitment, but did not improve glomerular or interstitial histopathology or survival (195).

In humans, phase 2 clinical trials were promising in diabetic kidney disease. A selective inhibitor of CCR2 (CCX140-B) added to standard care (196) and the Spielgelmer NOX-E36 showed evidence of kidney protection in patients with type 2 diabetes and kidney disease (197). However, there are no current ongoing phase 3 trials. In this regard, the standard of care has been changed by the efficacy of sodium-glucose cotransporter-2 (SGLT2) inhibitors and any future trial should test the efficacy of new drugs on top of SGLT2 inhibition and renin-angiotensin system (RAS) blockade (198). On the other hand, there are ongoing phase 2 trials of CCX140-B (Ilacirnon) for FSGS (NCT03536754, NCT03703908).

## Polarization of M1 to M2 Macrophages in Experimental Kidney Disease

Inducing macrophage polarization has been also proposed as a potential approach to reduce the kidney inflammatory response (21, 199). Strategies to induce macrophage polarization, that include stimulation with cytokines, miRNAs regulation or genetic manipulation (199), have been extensively investigated in preclinical studies to reduce the inflammatory response in kidney diseases.

### Cytokine-Induced Polarized Macrophages

Cell stimulation with different cytokines has been used to induce macrophage phenotype polarization and polarized cells were subsequently administered to treat experimental kidney disease (199). Administration of spleen derived macrophages (SPDM) polarized to M2 phenotype by IL-4/IL-13 stimulation *ex vivo* decreased histological and functional kidney injury as compared to M1 macrophages in experimental adriamycin nephropathy (AN) in immunodeficient mice (200). Likewise, transference of M2 macrophages generated *ex vivo* by incubation of SPDM with IL-10/TGF-β1 decreased kidney inflammation, structural injury and functional decline in the same model (201). Similarly, the administration of M1 macrophages induced by incubation with INF-γ increased tubular injury in murine IRI while M2 macrophages induced by incubation with IL-4 did not (59). In STZ-induced diabetic mice, transfusion of SPDM polarized to M2 by incubation with IL-4/IL-13 decreased tubular atrophy, glomerular hypertrophy, interstitial expansion and kidney fibrosis (202). In murine nephrotoxic nephritis, the transfusion of bone marrow-derived macrophages (BMDM) polarized to M2 phenotype (CD206^+^) by e*x vivo* incubation with IL-4/IL-13 reduced kidney injury, proteinuria, and glomerular inflammatory cell infiltration (203). Treatment with both BMDM polarized *ex vivo* to M2 macrophages by IL-4/IL-13 as well as *in vivo* M2 polarization induced by IL-4/IL-13 injections, reduced renal crystal formation in murine experimental kidney stone disease (204). A more recent study corroborated the beneficial effects of M2 transplantation in AKI. Peritoneal macrophages isolated from mice under peritoneal dialysis were polarized to M2 by incubation with IL-4/IL-13 and then injected into the renal cortex of mice with experimental ischemia reperfusion injury (IRI). M2 macrophages administration improved kidney injury and decreased inflammation compared to those injected with non-activated (M0) macrophages (205). Finally, the intrarenal administration of an Elastin-like Polypeptide (ELP-VEGF) construct induced a clear polarization to M2 macrophage phenotype in CKD in pigs and improved renal hemodynamics and fibrosis, despite no differences in renal macrophage infiltration were found compared to control (206).

### Polarized Macrophages Induced by Genetic Manipulation

Macrophages have been used successfully in cell therapy to deliver targeted therapeutic genes in models of inflammatory kidney disease. The adoptive transfer of bone-marrow-derived macrophages (BMDM) genetically modified with adenoviral particles encoding the anti-inflammatory cytokine interleukin 1 receptor antagonist (IL-1Ra) reduced kidney injury in murine anti-GBM (207). Similar results were observed using the same approach in unilateral ureteral obstruction (UUO) mice: the transference of macrophages encoding IL-1Ra after ureter obstruction reduced kidney interstitial macrophage infiltration, overall inflammation and fibrosis (208). In the same way, rat alveolar macrophages (NR8383) transfected with an adenovirus encoding IL-4 reduced albuminuria and glomerular macrophage infiltration (209). The delivery of IL-10-expressing macrophages or a dominant-negative I-KB in rat nephrotoxic nephritis also reduced kidney injury (210, 211). In murine kidney ischemia reperfusion injury (IRI), the transference of adenoviral transduced BMDM encoding Heme oxygenase-1 (HO-1)preserved kidney function (212). Similar results were observed in a rat kidney IRI. Herein, transference with adenoviral-IL-10-transduced BMDM markedly reduced albuminuria, the number of pro-inflammatory macrophages and fibrosis (210).

### Macrophage Polarization Induced by miRNAs

MicroRNAs (miRNAs) are small, single-stranded non-coding RNAs (20–24 nucleotides) that regulate post-transcriptional gene expression (213). Since their discovery, multiple potential roles have been attributed to these molecules, including their participation in the inflammatory response by regulating multiple processes, including macrophage phenotype polarization (214). The miRNA expression profiles in M1 and M2 macrophages have been characterized both in human and in murine macrophages (215–217) (Table 1). A recent review highlighted that miR-9, miR-127, miR-155, and miR-125b promote M1 polarization while miR-124, miR-223, miR-34a, let-7c, miR-132, miR-146a, and miR-125a-5p induce the M2 phenotype in both species (214). The relevance of some of these miRNAs in the macrophage phenotype context has been also corroborated in kidney disease with different results. miR-146a regulated local chronic inflammation in autoimmune glomerulonephritis in B6.MRLc1 mice, suggesting that it is a potential therapeutic target (224). However, genetic miR-146a deletion increased proteinuria, renal macrophage infiltration, glomerular hypertrophy and fibrosis in murine streptozotocin-induced diabetes (225). The anti-inflammatory effect of miR-146 through regulation of IL-6 expression was also demonstrated in macrophages derived from cystic fibrosis patients (222). Similarly, a transwell assay using glomerular mesangial cells from LN patients stimulated with a miR-146 mimic showed a reduction in macrophage migration (223). In the same way, although miR-9 has been reported to promote pro-inflammatory macrophage phenotype, mice transfected with lentiviral vectors expressing miR-9-5p were protected from UUO-induced kidney fibrosis through decreased infiltrating monocytes/macrophages and induction of metabolic reprogramming (218).

**Table 1:** MicroRNA and macrophage polarization.

**MicroRNA**	**Polarization**	**References**
miR-9	M1	(214)
miR-127		(214)
miR-155		(214)
miR-125b		(214)
miR-9-5p		(214, 218)
pre-miR21		(219)
miR-199a-5p		(220)
miRNA-19b-3p		(221)
miR-124	M2	(214)
miR-223		(214)
miR-34a		(214)
miR-132		(214)
miR-125a-5p		(214)
miR-146a		(214, 222, 223)
mature miR-21		(219)

miR-21 is a key mediator of the anti-inflammatory response in macrophages and has been proposed to exert a dual role. Pre-miR21 polarizes macrophages to the M1 phenotype while mature miR-21 polarizes to M2 and upregulates anti-inflammatory targets, such as IL-10 (219). However, although miR-21 has been widely studied in inflammatory conditions, its role in experimental kidney disease reported controversial results. Thus, while genetic deletion of miR-21 reduced kidney fibrosis in murine UUO, IRI, Alport syndrome (226, 227) and polycystic kidney disease (228), miR-21 was also reported to protect from murine kidney IRI (229). Lademirsen sodium (RG 012), an anti–miR-21, is currently undergoing phase 2 clinical trials for Alport syndrome (NCT02855268).

Apart from the miRNAs expressed directly in macrophages, miRNAs from extracellular vesicles also influence the macrophage polarization process. For example, miR-199a-5p present in extracellular vesicles from albumin-induced tubular epithelial cells promoted the M1 macrophage phenotype in mice with high fat diet (HFD)/STZ-induced diabetes (220). Similar results were obtained in another recent study demonstrating that miRNA-19b-3p from tubular epithelial-derived exosomes promotes M1 macrophage activation in a murine endotoxemia-induced AKI (221).

## Conclusion

Macrophages actively participate in the initial phases on kidney injury, as well as in kidney damage resolution and progression. Current pharmacological treatments that present beneficial effects in human CKD diminish macrophages infiltration in the kidney (1), including the SGLT2 inhibitors (230). Future investigations targeting macrophage polarization, and macrophage-derived cytokines could provide novel therapeutic approaches to further reduce the inflammatory response in kidney diseases.

In spite of the intensive research done, the lack of consistent nomenclature, and reliable polarization markers that are conserved between species and between *in vivo* and *in vitro* models of macrophage polarization has delayed progress in the field and complicates the extrapolation of preclinical results into human kidney disease (53). Moreover, it is necessary to develop preclinical models that truly resemble the human situation, something specially relevant in human kidney diseases due to the complex etiology of CKD (1). Some studies have been focused on comparative biology approaches on macrophages to accelerate the translation into the clinic (231). Moreover, individual variation in cytokine production in the human population has been reported (232). Novel data on transcriptional machinery of different macrophage subtypes have increased the knowledge about macrophage functions (233, 234). However, the mechanisms of genetic regulation remains unraveled. Future studies using cutting-edge technologies, including single-cell RNA sequencing, genomics, and proteomics approaches, as well as spatial transcriptomics in human biopsies are needed to improve the understanding of cytokine profiles, macrophage phenotypes and functions in different human diseases, including AKI, AKI-to-CKD transition, and CKD progression.

## Author Contributions

LT-S and AT-M contributed to the design of the figures. MO, LM-E, LS-S, and EC-N participated in the development of mouse models and analysis of data of the unpublished data and to the draft of some parts of the manuscript. EC-N, SR-M, TB, AS, AT-M, VM, and RR-D contributed to the draft of some parts of the manuscript. JE and AO contributed to the critical review of the manuscript and the financial support of the work. MR-O contributed to the draft of the manuscript and financial support. All the authors have reviewed the manuscript and approved the final version.

## Conflict of Interest

The authors declare that the research was conducted in the absence of any commercial or financial relationships that could be construed as a potential conflict of interest.
